# Cecal Leiomyosarcoma Management: A Case Report and Review of Literature

**DOI:** 10.7759/cureus.100334

**Published:** 2025-12-29

**Authors:** Lucas N Canaan, Ella N Lahart, Daniel Sarmiento

**Affiliations:** 1 Surgery, Northeast Georgia Medical Center Gainesville, Gainesville, USA; 2 Colorectal Surgery, Piedmont Hospital, Atlanta, USA

**Keywords:** colon leiomyosarcoma, colorectal cancer, lower gi or colorectal surgery, minimally access surgery, primary colorectal cancer

## Abstract

Leiomyosarcomas are very rare soft tissue sarcomas originating from smooth muscle cells. This neoplasm can develop in various places around the body, including but not limited to the uterus, retroperitoneum, colon, blood vessels, and bladder. This case report details the discovery of a cecal leiomyosarcoma in a patient who presented to the emergency department after a year of abdominal pain, fatigue, and unexpected weight loss. The report will discuss the available literature on leiomyosarcoma, the surgical approach for management in this patient, and post-operative management.

## Introduction

Leiomyosarcomas are rare soft tissue sarcomas originating from smooth muscle cells. When arising in the gastrointestinal (GI) tract, these tumors typically originate from the muscularis propria layer of the bowel wall [1]. They account for approximately 0.1% of all colorectal malignancies [2]. Due to their rarity, most of the existing literature consists of case reports and small case series.

One study by Yasinazi et al. analyzed 191 cases of colorectal leiomyosarcoma to better understand demographic trends [2]. The study found the highest prevalence among Caucasian patients aged 60 to 69, with a nearly equal distribution between males and females. The overall five-year survival rate was 50.3%, whereas five-year survival among patients who underwent resection was higher at 60.8%. Negative prognostic indicators included high tumor grade (Grade III and IV), regional spread, and distant metastases.

As highlighted in several case reports [3,4], the limited availability of data makes both diagnosis and treatment clinically challenging. Within the GI tract, leiomyosarcomas most commonly occur in the small intestine, with involvement of the colon and rectum being less frequent [5].

A critical diagnostic step is distinguishing leiomyosarcoma from gastrointestinal stromal tumors (GISTs). Before the identification of *KIT* (CD117) as a diagnostic marker in 1998, many mesenchymal tumors were misclassified as leiomyosarcomas. With the discovery that *KIT *is expressed in GISTs but not in leiomyosarcomas, the reported incidence of leiomyosarcoma significantly declined [1]. This distinction is vital, as treatment strategies differ significantly between the two entities. The implication for the distinction between a GIST and leiomyosarcoma is the overall management strategy. Although surgery is commonly the first-line treatment for both conditions, management strategies evolve as tumors increase in size or grade. Large or high-grade gastrointestinal stromal tumors are often first treated with neoadjuvant imatinib, followed by surgical resection without lymphadenectomy, whereas leiomyosarcomas are generally managed with surgical excision, with radiation or chemotherapy reserved for selected cases [1].

This case report presents the clinical presentation, surgical management, and postoperative course of a 57-year-old woman ultimately diagnosed with a cecal leiomyosarcoma.

## Case presentation

A 57-year-old woman initially presented to the emergency department with periumbilical abdominal pain that had persisted for at least one year. Her medical history was significant for hyperlipidemia, hypertension, and rheumatoid arthritis. Over the course of the year, she also experienced increasing fatigue and weakness, diarrhoea, and unintentional weight loss.

As part of her evaluation, the patient underwent both a colonoscopy and an esophagogastroduodenoscopy (EGD). Colonoscopy findings included small-mouthed diverticula in the sigmoid colon and diffuse moderate inflammation throughout the colon. The inflammation was characterized by altered vascularity, congestion (edema), erosions, erythema, friability, loss of vascular pattern, and confluent ulcerations. No explicit comment of a mass was noted. Biopsies taken during the procedure revealed chronic active colitis with severe activity.

The EGD showed a 2 cm hiatal hernia and mild inflammation in the gastric antrum, marked by erosions and erythema. Biopsies from these areas did not reveal any abnormal pathology.

**Figure 1:**
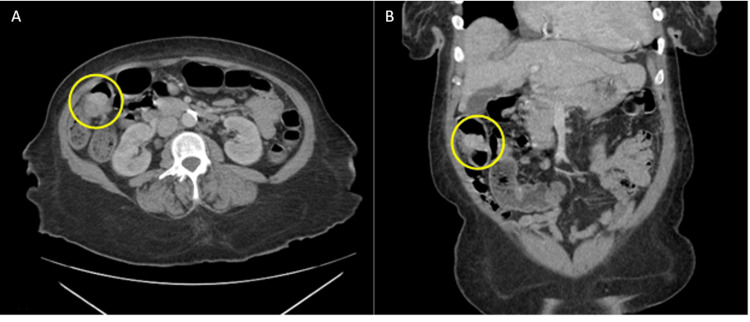
(A) CT imaging of the abdomen and pelvis in axial views demonstrating the cecal mass in question highlighted by the yellow circle. (B) CT imaging of the abdomen and pelvis in coronal views demonstrating the ileal mass in question highlighted by the yellow circle.

The patient ultimately returned to the hospital one week prior to this presentation due to a severe worsening of her abdominal pain. In the emergency department, she was found to have severe blood loss anemia, with a hemoglobin level of 3.8 g/dL. Computed tomography (CT) imaging of the abdomen and pelvis revealed a mass in the terminal ileum concerning for a neoplasm measuring approximately 3 cm, which can best be seen in Figure 1.

Additional laboratory findings are summarized in Table 1. On examination, her vital signs were notable for tachycardia but were otherwise within normal limits. 

**Table 1:** Initial laboratory findings.

Lab values	Measured values	Range
White blood cell (WBC)	15.87	3.00-10.80
Hemoglobin (Hgb)	3.8 g/dL	12.00-16.00 g/dL
Hematocrit (Hct)	15.2 %	37.3-47.4 %
Platelets (Plt)	783	136-407
Sodium	140 mmol/L	136-145 mmol/L
Potassium	3.4 mmol/L	3.5-5.1 mmol/L
Chloride	109 mmol/L	99-109 mmol/L
CO_2 _	22 mmol/L	22-31 mmol/L
Blood urea nitrogen (BUN)	12 mg/dL	6-18 mg/dL
Creatinine	0.73 mg/dL	0.55-1.02 mg/dL
Glucose	93 mg/dL	60-110 mg/dL

The patient was resuscitated with blood products, resulting in improvement of both her hemoglobin levels and tachycardia. Due to the complexity of her condition and acute presentation, she was transferred to a higher level of care. Upon arrival at the receiving hospital, the colorectal surgery team was consulted for further management. Given her ongoing transfusion requirements and the presence of a mass on imaging, the decision was made to proceed with a robot-assisted right hemicolectomy with primary ileocolonic anastomosis.

Intraoperative findings revealed a large cecal mass with extensive lymphadenopathy along the ileocolic trunk and the left branch of the middle colic artery. These intraoperative findings were consistent with the preoperative CT imaging of the abdomen and pelvis. There was no evidence of peritoneal spread or metastatic implants involving other visceral organs. The procedure was performed using a robot-assisted laparoscopic approach. Dissection was carried out in a medial-to-lateral fashion, with high ligation of the ileocolic trunk using a surgical stapler after proper identification and protection of the duodenum. The avascular plane between the mesentery, colon, and retroperitoneum was developed to the hepatic flexure and laterally toward the abdominal wall. Remaining retroperitoneal attachments were divided in a distal-to-proximal fashion, starting at the hepatic flexure and extending along the white line of Toldt, including pelvic attachments of the terminal ileum.

The proximal transection point was selected on the ileum and divided with a surgical stapler; the distal transection point was similarly made on the transverse colon. An intracorporeal, isoperistaltic ileocolonic anastomosis was then performed. The specimen was removed and sent to pathology for evaluation.

**Figure 2:**
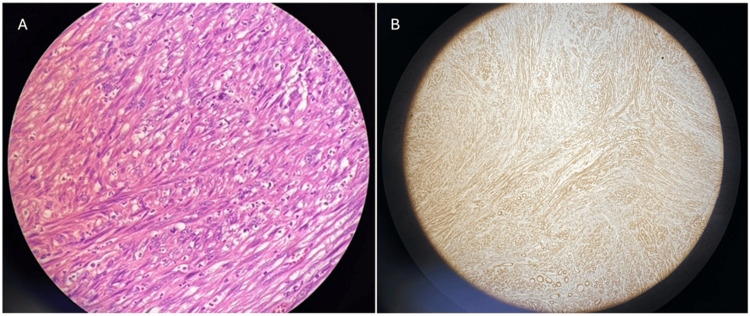
(A) H&E stain of a colonic leiomyosarcoma specimen. (B) Smooth muscle actin (SMA) staining of specimen positive for smooth muscle consistent with a leiomyosarcoma.

Pathologic evaluation revealed a Fédération Nationale des Centres de Lutte Contre le Cancer (FNCLCC) grade 2 (G2) leiomyosarcoma. The tumor was confined to the cecum, measuring 2.8 cm at its greatest dimension. All surgical margins were negative, with the closest margin measuring 5.2 cm. A total of 22 lymph nodes were examined with no signs of metastatic disease. The mitotic rate of 5-9 mitoses per 10 high-power fields (HPF), and necrosis was noted to be present, with less than 5%. The pathologic stage was determined to be pT1pN0. Given the rarity of this tumor, a second pathological review was requested, which confirmed the initial findings. Histology from the specimen can be seen in Figure 2 below.

Postoperative recovery was uneventful. The patient did not develop postoperative ileus and was able to tolerate a diet in the immediate postoperative period without complications. Following tumor resection, her hemoglobin stabilized and improved without further transfusions. She was discharged on postoperative day three with no surgical complications documented in the medical record.

During her inpatient stay, a CT scan of the chest, abdomen, and pelvis was performed and showed no evidence of metastatic disease. No additional staging imaging was deemed necessary. One comment from the chest CT was a ground-glass opacity in the left upper lobe. The plan for this abnormality is to follow up with a repeat scan in three months. The patient was referred to medical oncology to discuss adjuvant therapy. As the tumor was localized to the colon, node-negative, and without evidence of metastasis, no chemotherapy or radiation therapy is currently planned. Additional molecular testing of the tumor has been requested to identify any targetable mutations in the event of future recurrence. The surveillance plan includes a CT scan of the chest, abdomen, and pelvis with contrast on a yearly basis, and a colonoscopy one year after surgery.

## Discussion

Colonic leiomyosarcoma is a rare and aggressive mesenchymal malignancy, accounting for less than 0.1% of all colorectal cancers. Its rarity often results in delayed diagnosis and challenges in developing standardized treatment guidelines [6]. The present case of a 57-year-old woman with a 2.8 cm, grade 2 colonic leiomyosarcoma exemplifies an early-stage presentation, which is unusual, as most cases are discovered at more advanced stages. Histologically, colonic leiomyosarcomas are characterized by smooth muscle differentiation and are immunohistochemically positive for SMA and desmin, with negative staining for CD117 (KIT), DOG1, and CD34, which distinguishes them from gastrointestinal stromal tumors (GISTs) [6]. This differentiation is crucial, as GISTs are typically sensitive to tyrosine kinase inhibitors, while colonic leiomyosarcomas are not. Misclassification was common before the discovery of KIT, which significantly refined the categorization of gastrointestinal mesenchymal tumors [1].

The standard treatment remains complete surgical excision with negative margins [1]. In a large national cancer database (NCDB) review by Cooper et al., among 433 patients with colorectal sarcomas, those who underwent surgical resection had significantly better outcomes, with the five-year overall survival rate improving from 43.8% to over 70% in localized cases [7]. Additionally, lymphadenectomy has unclear prognostic value in colonic leiomyosarcoma, given its hematogenous spread, but was shown to benefit patients when at least 13 nodes were harvested. This case met the benchmark, with 22 reactive, non-malignant lymph nodes examined.

Adjuvant therapy remains controversial and is generally reserved for high-grade or metastatic leiomyosarcoma. A retrospective European cohort study by Savina et al. found limited benefit from anthracycline-based chemotherapy in early-stage or low-grade tumors, though it was used in metastatic or high-risk cases [8]. Similarly, radiation therapy may reduce local recurrence in rectal sarcomas but has not been shown to improve survival in colonic leiomyosarcoma. Accordingly, in this case, the patient's tumor was small, low-grade, and completely resected, so adjuvant treatment was not administered, which is consistent with current recommendations. Prognostically, tumor size, grade, and metastatic spread are key factors. In a multicenter study by Zagami et al., patients with localized gastrointestinal leiomyosarcoma had a five-year overall survival rate of 73%, compared to a median survival of just 16.4 months in metastatic disease [9]. Tumors under 5 cm and patients under 60 years of age generally fared better. Our patient fits this low-risk category and demonstrates excellent short-term postoperative recovery without recurrence or complications.

Despite favorable pathology, long-term surveillance is essential due to leiomyosarcoma’s potential for late recurrence. Guidelines suggest imaging every 3-4 months during the first two years, then gradually extending the interval. The case series by Doan et al. underscores this need: one patient developed peritoneal metastasis within eight months of surgery, while another remained disease-free with routine follow-up alone [10]. This case illustrates a rare but clinically significant malignancy managed according to the best available evidence. The patient’s tumor biology, early detection, complete surgical excision, and absence of metastasis position her for a favorable prognosis, though continued surveillance is critical given the unpredictable nature of leiomyosarcoma.

## Conclusions

This case represents a classic clinical presentation of colon cancer, ultimately diagnosed as a rare pathology, colonic leiomyosarcoma. Although uncommon, this entity was managed according to established oncologic principles, including surgical resection and appropriate postoperative care. In this case, the patient was fortunate to have a tumor that was completely excised with no metastatic spread or lymph node involvement, requiring no further treatment, and the patient has shifted into an observation pathway. While adenocarcinoma remains the predominant histologic subtype of colorectal malignancy, rare tumors such as leiomyosarcoma do occur and warrant consideration. This case highlights the importance of including leiomyosarcoma in the differential diagnosis of colonic masses to ensure accurate diagnosis and optimal management.
